# pSTAT3: a target biomarker to study the pharmacology of the anti-IL-21R antibody ATR-107 in human whole blood

**DOI:** 10.1186/1479-5876-11-65

**Published:** 2013-03-14

**Authors:** Ming Zhu, Susan Pleasic-Williams, Tsung H Lin, David A Wunderlich, John B Cheng, Jaime L Masferrer

**Affiliations:** 1 Precision Medicine, Pfizer BioTx Clinical R&D, 200 Cambridge Park Drive, Cambridge, MA 02140, USA; 2 Regulated Bioanalysis and Biotherapeutics Clinical Research, Pfizer Worldwide R&D, Groton, CT, USA; 3 Immunoscience, Pfizer Worldwide R&D, Cambridge, MA, USA

**Keywords:** Biomarker, Target engagement, Mechanism of action, IL-21, pSTAT3, ATR-107, IL-21R

## Abstract

**Background:**

IL-21 has been shown to play an important role in autoimmune diseases. ATR-107 is an antibody which directly targets the IL-21 receptor (IL-21R). To aid the clinical development of ATR-107, there is a need for understanding the mechanism of action (MOA) of this antibody when assessing target engagement in human subjects.

**Methods:**

To determine ATR-107 biological activity and potency in human blood, its inhibitory function against IL-21 induced STAT3 phosphorylation in human peripheral T and B cells was measured.

**Results:**

The data show that IL-21 induces STAT3 phosphorylation in a concentration-dependent manner, consistent with its migration to the nuclear. Using a flow cytometry based functional whole blood assay, ATR-107 is demonstrated to be a potent IL-21 pathway inhibitor. It competes with IL-21 for receptor binding in a competitive manner, but once it binds to the receptor it behaves like a non-competitive inhibitor, most probably due to the long observed k_off_. The concentration-dependent inhibition observed with ATR-107 correlates inversely with the levels of receptor occupancy, both in *ex vivo* whole blood assays and directly in human blood when ATR-107 was given to healthy volunteers.

**Conclusions:**

IL-21 induced phosphorylation of STAT3 in T and B cells can be used as a biomarker to evaluate the target engagement of ATR-107 in human whole blood. The antibody behaves like a potent non-competitive inhibitor blocking IL-21 induced STAT3 phosphorylation for a long period of time. These results may help with the translation of preclinical information and dose selection towards ATR-107 clinical efficacy.

## Introduction

Interleukin-21 (IL-21) is a recently discovered type I cytokine that modulates the proliferation and function of various cell types, including T cells, B cells and NK cells [1]. It also contributes to the development of the Th17 cell lineage [2].

IL-21 signals through a heterodimeric receptor complex consisting of a shared common gamma chain (γc), and a high affinity IL-21R alpha chain [3]. IL-21 is mainly produced by activated CD4^+^ T cells and by natural killer T (NKT) cells [4]. The IL-21 receptor however, is expressed in most lymphoid, hematopoietic cells, fibroblasts, keratinocytes and intestinal epithelial cells [5]. Once IL-21 binds to its receptor, it activates JAK1 and JAK3 tyrosine kinases, which in turn phosphorylate STAT1, STAT3 and STAT5 and initiate the transcription of regulated genes [6,7]. IL-21 modulates adaptive immune responses by its affect on broad range of immune cells, including T, B and NK cells [3]. Preclinical data has revealed the importance of this cytokine in several autoimmune diseases. For example, *in vivo* data show that blockade of IL-21 signaling using an IL-21 receptor Fc fusion protein (IL-21R Fc) decreases the disease severity in several murine models including collagen-induced arthritis [8], the MRL-Fas^lpr^ lupus model [9] and the diabetic NOD model [10]. The shared mechanism in these autoimmune models appears to be the pathophysiological role of IL-21 effects on cytokine and autoantibody production.

The use of biomarkers in drug development is very important in understanding the mechanism of action, dose selection and patient stratification. Since STAT3 is a direct downstream signal of IL-21R activation, and it plays a critical role in regulating immune responses [3,4,11-13], we sought to use STAT3 phosphorylation as a new pharmacodynamic biomarker to understand the mechanism of action of ATR-107.

In order to block the IL-21 signaling pathway, a high affinity humanized antibody was developed to directly target both human (KD: 2.02 nM) and mouse (KD: 16.72 nM) IL-21R [14]. Previous studies showed that the antibody ATR-107 significantly reduces blood anti-dsDNA antibody level and kidney IgG deposits in the MRL-Fas^lpr^ mouse model of lupus [14]. Its pharmacokinetics and pharmacodynamic (PD) activity has also been evaluated in cynomolgus monkeys. Following a single iv dose of 10 mg/kg, the serum half-life (t_1/2_) was reported to be approximately 10 days [15]. Interestingly, in these animals, the PD effect lasted much longer, between 5 and 13 weeks, when measured by the IL-21 induced IL-2Rα gene expression [15,16]. The apparent disconnection between pharmacokinetic and pharmacodynamic of the antibody led us to investigate its mechanism of action and pharmacological efficacy in the human system. Thus, a series of experiments were carried out to determine the effects of ATR-107 on IL-21 induced STAT3 phosphorylation in human peripheral blood T and B cells. This assay was then used clinically to evaluate the pharmacodynamic effect of this drug in healthy volunteers.

## Material and methods

### Reagents

Recombinant human IL-21 (IL-21), ATR-107, human IgG triple mutant (IgG1 TM) were prepared by the Biotherapeutic Technologies Department (Cambridge, MA) at Pfizer.

Blood was drawn from 14 female and 13 male healthy volunteers (age 24–61 years) into heparinized collection tubes in accordance with Pfizer protocol (protocol #: GOHW RDP-01) approved by the Shulman Institutional Review Board.

### T cell purification

CD4^+^ T cells from healthy donor peripheral blood were isolated using RosetteSep® Human CD4^+^ T Cell Enrichment Cocktail (cat#: 15062) from STEMCELL Technologies Inc. (Vancouver, Canada), according to the manufacturer’s instruction. Briefly, RosetteSep® Human CD4^+^ T Cell Enrichment Cocktail was added to the blood at concentration of 50 μL/mL blood and incubated for 20 minutes at room temperature. Then the samples were diluted with an equal volume of PBS + 2% FBS and layered on top of Ficoll-Paque™ PREMIUM density medium (Piscataway, NJ). After centrifugation at room temperature for 20 minutes at 1200 x g, cells at the plasma-Ficoll interface were harvested and washed twice with PBS + 2% FBS. Cells were counted using Cellometer auto T4 cell counter (Nexcelom Bioscience, Lawrence, MA).

### T cell stimulation

T cells were resuspended in PBS + 2% FBS and aliquoted into 96 well plates at 1x10^6^ cells/100 μl/well. ATR-107 at various concentrations was added for 30 minutes before IL-21 stimulation. 10 ng/ml IL-21 was used and the stimulation was stopped 15 minutes later by adding 3X loading buffer (Cell Signaling Technology, Inc., Danvers, MA) plus proteinase inhibitor and phosphatase inhibitor cocktail (Sigma-Aldrich Corp. St. Louis, MO). Samples were then transferred to 1.5 ml Eppendorf tubes and kept on ice. Fifteen seconds of sonication was used to shear the DNA and reduce viscosity of the samples.

### Western blot

Sonicated samples were heated at 95°C to denature the proteins, followed by a quick spin in a desktop centrifuge. 30 μl of the samples were then loaded and proteins were separated using a 10% SDS-PAGE Tris-glycine gel (Invitrogen, Grand Island, NY). Transfer was done with the iBlot® Gel Transfer system (Invitrogen) onto a nitrocellulose membrane. Blocking was done with PBS + 5% non-fat milk powder (Bio-Rad Laboratories, Hercules, CA) for one hour. Then the membrane was rinsed three times with PBS + 0.1% Tween-20, and incubated with the primary antibodies at 4°C overnight in PBS + 5% BSA. pSTAT3 (Tyr705, Cat#: 9145 s) and STAT3 (Cat#: 9139 s) antibodies (Cell Signaling Technology) were used at 1:1000 dilution.. HRP-conjugated secondary antibody was used at 1:10,000 dilution and incubated with the membrane at room temperature for one hour. Signal was visualized with Visualizer™ Western Blot Detection Kit (Millipore, Billerica, MA) using AlphaImager™ 3400 system (Alpha Innotech) and FluorChem 5500 software (Alpha Innotech).

### pSTAT3 whole blood assay

Assay was done as previously described [17] with slight modifications. Briefly, blood from healthy volunteers was drawn with heparin as the anti-coagulant, and assayed within 2 hours after blood collection. Blood was aliquoted into 100 ul/ well in 96 well deep well plates and incubated at 37°C for 30 minutes with the indicated amount of ATR-107 or control Ab (hIgG1-TM). Blood was then treated with 1 – 1000 ng/ml of IL-21 for 15 minutes at 37°C, followed by addition of 10 volumes of 1X lyse/fix solution to stop the reaction*.* Anti-CD4 Pacific Blue and anti-CD19 FITC (BD Biosciences) were added 15 minutes before the IL-21 for surface staining for a total of 30 minutes. Samples were washed twice by centrifugation using PBS containing 2% heat inactivated FBS, then permeabilized with 80% ice cold methanol for 30 minutes. Alexa Fluor 647 (A647) labeled anti-pSTAT3 (Tyr705) antibody (BD Biosciences, cat#: 557815) was then added and incubated at 4°C overnight. Data was collected the next day on FACS Calibur (BD Biosciences) or LSRFortessa (BD Biosciences) and analyzed using Flowjo software (version 7.6.1, Tree Star, Inc. Ashland, OR).

### Target coverage

To understand the level of ATR-107/IL-21R binding, an Alexa Fluor 647 dye was conjugated directly to ATR-107 (ATR-107-A647) with a dye to antibody ratio of 3:1 to 5:1. To evaluate the competition between ATR-107-A647 (3.6 ug/ml) and IL-21, these two reagents were added to the samples at the same time, and the reaction was stopped after 30 minutes using 10 volumes of 1X Lyse/Fix buffer. The remaining cells were washed twice with PBS containing 2% heat inactivated FBS. Samples were then subjected to data collection using FACS Calibur (BD Biosciences) or LSRFortessa (BD Biosciences) without fixation, permeabilization and intracellular staining. Maximum binding was determined by incubating samples with only ATR-107-A647. Non-specific binding (NSB) was determined by incubating samples with ATR-107-A647 in the presence of excessive amount of unconjugated ATR-107 (363 ug/ml). Non-specific binding (NSB) was subtracted from each data point and the resulting value expressed as a percentage of maximum binding.

To compare the target engagement and functional effects on the signaling, the receptor occupancy assay was done side-by-side with the pSTAT3 assay. In those experiments, the receptor occupancy assay was performed similar to the pSTAT3 assay except that A647-ATR-107 was added to the samples, instead of IL-21.

### Imaging flow cytometry

To validate functional response, the translocation of pSTAT3 to the nucleus of CD4^+^ T lymphocytes in response to IL-21 was determined using an Amnis ImageStream 100 (Amnis Corporation, Seattle WA). The method to prepare samples for imaging analysis was similar to the aforementioned pSTAT3 whole blood assay, except that the nuclear dye DAPI (4.5 μM final concentration) was incubated with permeabilized and stained leukocytes prior to cellular acquisition by the ImageStream. The data were analyzed with a nuclear translocation analysis wizard in the IDEAs software (Amnis Corporation), as previously described [18]. Briefly, single focused leukocytes were identified and then followed by gating DAPI- and A647-double positive events. Nuclear translocation of pSTAT3 in CD4^+^ events was measured by a nuclear morphology mask to assess a similarity score of the correlation of pixel values between DAPI and A647-pSTAT3 images on a per-cell basis.

### Data analysis

The percent of maximum pSTAT3 was determined by subtracting the background (autofluorescence) value from each data point. This corrected value was then divided by the maximum response for a given dose of IL-21 and multiplied by 100.

### Statistical analysis

Data from different donors were pooled together using the percentage of maximum activation described above, and the IC50 was calculated using GraphPad Prism software version 5.02 (La Jolla, CA) using least square fit analysis.

## Results

### ATR-107 inhibits IL-21 induced phosphorylation and nuclear translocation of STAT3

**Figure 1:**
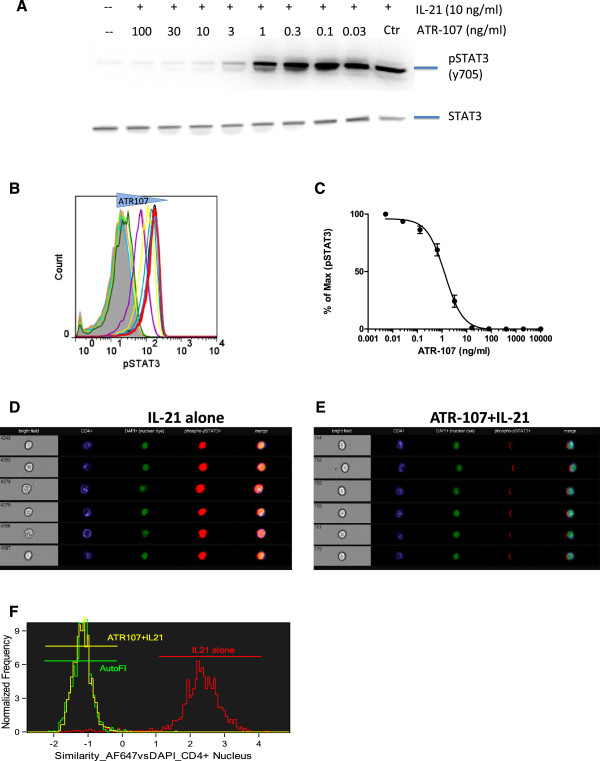
**ATR-107 concentration-dependently inhibited IL-21 induced STAT3 phosphorylation. A**: Purified T cells from healthy donor peripheral blood were pre-incubated with ATR-107 at concentrations from 0.03 ng/ml to 100 ng/ml or control antibody for 30 minutes, followed by stimulation with IL-21 (10 ng/ml) for an additional 15 minutes. Total and phospho–STAT3 was detected by Western blot. **B**: Healthy donor whole blood was pretreated with ATR-107 or control Ab for 30 minutes prior to stimulation with 10 ng/ml IL-21 for 15 minutes. Cells were fixed, permeabilized, stained with A647-labeled anti–phospho–STAT-3 antibody, and analyzed using flow cytometry gated on CD4^+ ^T cells. Representative histogram showing the baseline (gray area), IL-21 + control Ab (red line) and IL-21 with various amount of ATR-107. **C**: Inhibition of ATR-107 on pSTAT3 mean fluorescence intensity (MFI) induced by 10 ng/ml IL-21(n = 6). **D**: Representative image of 6 individual cells detected by ImageStream cytometer (ISC) after treatment with 10 ng/ml IL-21 for 15 minutes. In addition to the bright field image (left column), cells were stained with CD4 (blue), DAPI (green) and pSTAT3 (red). Merged imaging shows pSTAT3 staining overlap with the nuclear (DAPI) staining (far right column). **E**: Translocation of pSTAT3 was completely blocked by pre-treatment of the samples with ATR-107 (4.3 ug/mL) for 30 minutes. **F**: Overlay of similarity of nuclear pixel images of DAPI and AF647 dyes in CD4^+^ T cells. Autoflurorescence (AutoFl) was a measurement of the sample treated with neither ATR-107 nor IL-21. The data were analyzed by the IDEAs software (Amnis Corporation).

Even though IL-21 activates several STAT proteins including STAT1, STAT3, STAT5a and STAT5b, the activation of STAT3 was studies since it is the most significant and sustained for this pathway [3]. To demonstrate that ATR-107 blocks the function of IL-21, CD4^+^ T cells, isolated from healthy donor peripheral blood, were stimulated with IL-21 for 15 minutes in the presence of various concentrations of ATR-107. Phosphorylation of STAT3 was visualized by Western blotting using an antibody specifically against pSTAT3 (Tyr705) (Figure 1A). IL-21 strongly induced phosphorylation of STAT3, while it didn’t change the expression of total STAT3. This IL-21-induced phosphorylation of STAT3 was inhibited by ATR-107 in a concentration-dependent manner, with maximal inhibition observed at or above a dose of 10 ng/ml.

The activity of ATR-107 was further evaluated when present in human blood using flow cytometry, a technology which is capable of sorting out cell populations of interest without the need for cell isolation. Phospho-STAT3 was measured using a specific antibody conjugated with fluorophore, thus allowing a quantitative analysis. Similar to isolated CD4^+^ T cells, phosphorylation of STAT3 was robustly induced by 10 ng/ml IL-21 in CD4^+^ T cells in whole blood as quantified by the intensity of the fluorescence, and ATR-107 concentration dependently inhibited IL-21-induced phosphorylation of STAT3 (Figure 1B). When levels of pSTAT3 (fluorescence intensity) were plotted against ATR-107 concentrations, an inhibition curve was generated indicating 50% of inhibition (IC_50_) at 1.45 ± 0.26 ng/ml, n = 6 (Figure 1C). Similar results were observed when CD19^+^ B cells were gated for analysis (data not shown).

Once phosphorylated, STAT3 forms dimers and translocates to the nucleus. We further examined the effect of ATR-107 on nuclear translocation of STAT3 using imaging flow cytometry. Cells were interrogated individually, fluorescence intensity recorded and images captured in imagestream cytometry. With the help of nuclear staining, the nucleus can be identified within the cell. As seen in Figure 1D, after IL-21 stimulation, an increase of phospho-STAT3 was detected (in red). Phospho-STAT3 was located in the same cellular compartment as the nuclear staining (in green). Merging of these two images (right column) confirms they overlap with each other. Pretreatment of cells with ATR-107 blocked phosphorylation of STAT3 and prevented STAT3 from translocating to the nucleus (Figure 1E) as no overlap of phospho-STAT3 and the nucleus was detected. Consistent with the images seen with individual cells, the nuclear translocation response to IL-21 in CD4^+^ cellular population, as shown in Figure 1F, was markedly increased and was exquisitely sensitive to ATR-107 pre-incubation. Collectively, these data shows ATR-107 blocks not only IL-21 induced STAT3 phosphorylation, but nuclear translocation in human CD4^+^ T cells.

### ATR-107 behaves like a non-competitive inhibitor for IL-21 signaling

**Figure 2:**
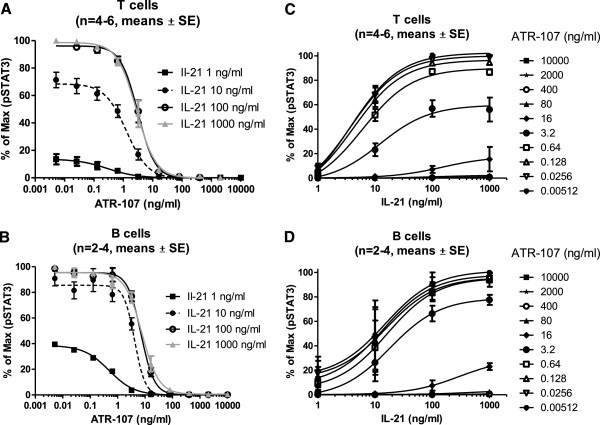
**ATR-107 behaves like a non-competitive antibody. **Summarized results of ATR-107 inhibition on STAT3 phosphorylation in peripheral T (**A**) and B (**B**) cells induced by different amount of IL-21. Whole blood was pre-incubated with various amounts of ATR-107, as indicated on the graphs, for 30 minutes, followed by 1–1,000 ng/ml of IL-21 stimulation for 15 minutes. The reaction was stopped, and the RBC removed by adding the BD Lyse/fix buffer. The remaining cells were then washed, permeabilized and stained for pSTAT3. Shown are the means ± SE from 2–3 separate experiments. % of Max was defined as: (MFI – background) / (Max response – background) X 100. Same data was re-plotted with IL-21 at the x-axis (**C**, **D**).

To characterize the inhibitory activity of ATR-107, inhibition curves for this molecule were generated using different amounts of IL-21. Pooled data from 4–6 donors showed that 15 minute incubation with increasing amounts (1 – 100 ng/ml ) of IL-21 led to a proportional increase in the levels of STAT3 phosphorylation in CD4^+^ T cells (Figure 2A). Maximal effect was found when cells were treated with 100 ng/ml of IL-21, since similar levels of pSTAT3 were observed with a dose of 1,000 ng/ml of IL-21. ATR-107 concentration-dependently inhibited the STAT3 phosphorylation at all doses of IL-21. However, the inhibition curves did not shift to the right in a parallel manner with increasing concentrations of IL-21. Furthermore, ATR-107 at the dose of 10 ng/ml almost completely blocked STAT3 phosphorylation, regardless of the IL-21 concentration that was used for stimulation (Figure 2A). These data suggest that ATR-107 is not functioning as a typical competitive inhibitor of IL-21. Similar results were observed when data was analyzed in CD19^+^ B cells (Figure 2B).

To better observe the mechanism of inhibition, the same data were re-plotted to show the IL-21 dose response curve, in the presence of different amounts of ATR-107 (Figure 2, C and D). Consistent with data shown in Figure 2A and B, IL-21 increased pSTAT3 level concentration dependently. However, when the ATR-107 concentration is higher than 0.64 ng/ml, the maximum stimulation could not be reached by 100 ng/ml or 1000 ng/ml of IL-21. This suggests the blockade of ATR-107 could not be overcome by high doses of IL-21, and indicates the non-competitive nature of this antibody.

### ATR-107 competes with IL-21 for receptor occupancy

**Figure 3:**
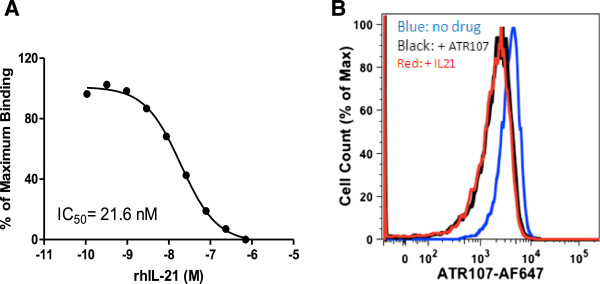
**ATR-107 competes with IL-21 for receptor binding on naïve B-cells (CD19**^**+**^**CD27**^**-**^**). A**: Increasing concentrations of IL-21 decreased binding of ATR-107-A647 to the naïve B-cells as measured by the A647 fluorescence using flow cytometry. Maximum binding was determined by incubating human blood with ATR-107-A647 (3.6 ug/mL) in the absence of IL-21. Non-specific binding (NSB) was subtracted from each data point and the resulting value expressed as a percentage of maximum binding. **B**: Representative flow cytometry histograms showing the overlay of the receptor occupancy performed without (blue line), or with either excessive concentration of IL-21 (707 nM, red line) or ATR-107 (363 ug/ml, black line). At least 2000 CD19^+^CD27^-^events were acquired for analysis.

To further characterize its mechanism of action, an IL-21R occupancy assay was developed [19]. For this purpose, ATR-107 was labeled with A647 and its binding to the IL-21R was tested in the presence of different amounts of IL-21. For this experiment, naïve B cells were chosen since they have high amounts of IL-21R (data not shown). Also, ATR-107-A647 was not pre-incubated with the samples. In other words, IL-21 and ATR-107-A647 were added at the same time to determine their initial rate of competitive binding (k_on_). As expected, the binding of ATR-107-A647 to naïve B cells decreased with increasing amounts of IL-21 with an IC_50_ of 21.6 nM (Figure 3). Similar results were observed using a commercially available IL-21 (eBioscience, San Diego, CA) (IC_50_ of 53 ± 23 nM, n = 3). This data suggest ATR-107 does compete with IL-21 for receptor binding when they were both added at the same time, regardless of the IL-21 source.

### Inhibition of ATR-107 on IL-21 induced pSTAT3 correlates with its receptor occupancy

**Figure 4:**
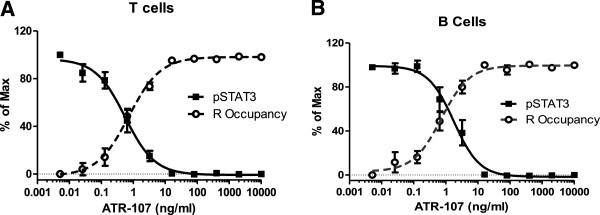
**ATR-107 inhibition on IL-21 induced STAT3 phosphorylation correlates inversely with IL-21R occupancy, *ex vivo *(A, B). **Receptor occupancy assays and pSTAT3 assays were performed side by side in T cells (**A**) and B cells (**B**). Briefly, whole blood samples were pre-incubated with ATR-107 for 15 minutes at 37°C, and stained with cell surface markers (CD4, CD19) for 15 minutes. Then either ATR-107-A647 or 10 ng/ml of IL-21 were added to the wells, and incubated at 37°C for another 15 minutes (for pSTAT3 assay) or 20 minutes (for receptor occupancy assay), followed by the addition of the Lyse/fix buffer to stop the reaction and to remove RBC. After centrifugation, the cell pellets were washed twice with PBS + 2% FBS. Samples were then collected with (for pSTAT3 assay) or without (for receptor occupancy assay) permeabilization and stained with pSTAT3 A647. Data shown are the means ± SE from 8 donors.

To further validate this assay, and to understand if the ATR-107 blockade measured by the functional pSTAT3 assay correlates with the receptor occupancy, these two assays were performed side by side in several different experiments. For this experiment, ATR-107 was pre-incubated for 30 minutes, and then either A647-labeled ATR-107 or IL-21 was added to assess the level of IL-21 receptor occupancy and STAT3 phsophorylation. As seen in Figure 4A for T cells and Figure 4B for B cells, an excellent inverse correlation was observed between the inhibition of the pSTAT3 signal and the occupancy of the IL21R by ATR-107. The receptor occupancy assay was then used to analyze blood samples obtained from healthy volunteers treated with a single dose of 60 mg of ATR-107. Complete receptor occupancy was observed for up to 42 days post-dose as reported by Pleasic-Williams et al. [19]. At the same time, the pSTAT3 response was determined by adding a dose of 10 ng/ml of IL-21 to the healthy volunteer blood. The pSTAT3 response in CD4^+^ T cells was inhibited by more than 90% in all treated subjects (n = 4) as compared to the signal generated in the placebo group (n = 3). This indicates again that these two assays correlate well with each other, and ATR-107 has a long pharmacodynamic effect *in vivo*[19].

## Discussion

One of the important strategies to understand the pharmacology of a new drug candidate is the use of biomarkers that can provide pharmacodynamic information from clinical samples regarding the interaction of the drug with its target. The effects of ATR-107, an antibody against the IL-21R, in suppressing IL-21 effects on immune cells were analyzed by measuring the effects of this antibody on the activation of STAT3. Other γc cytokines, such as IL-2, IL-7, and IL-15, predominantly phosphorylate STAT5 upon stimulation, while IL-21 predominantly phosphorylates STAT3. Additionally, STAT3 has a prolonged activation over other STATs and plays a critical role in the IL-21 signaling [3]. pSTAT3 measurements resulted in a very good dynamic range for flow cytometry analysis allowing the generation of excellent dose response curves and a good IC_50_ determinaion. Thus, phospho-STAT3 was selected as a biomarker for IL-21. Using Western blot, we clearly demonstrated the phosphorylation of STAT3 by IL-21 in T cells. The specificity of the pSTAT3 signal and, the inhibitory effects of ATR-107, were demonstrated by the concentration-dependent inhibition of pSTAT3 using Western Blot. To further our analysis into specific cell types, flow cytometry was used. Phospho-specific flow cytometry measures the phosphorylation state of the interested kinase(s) at a single cell level in a heterogeneous cell population [20]. Since the specific cell type can be identified by the simultaneous surface staining, there is no need to isolate the cells of interest. Another advantage is that the cells are kept in the whole blood environment during stimulation enabling the measurements in a more physiological condition.

Using flow cytometry analysis, pSTAT3 was detected in CD4^+^ T and CD19^+^ B cells. STAT3 signal transduction pathway is well known. After activation by phosphorylation, pSTAT3 translocates to the nucleus and transcription is initiated [3]. Indeed, when IL-21 was added to whole blood, a concentration- and time-dependent increase in pSTAT3 signal was observed that coincided with the movement of the transcription factor to the cell nucleus. This was clearly visualized using ImageStream in a single cell. In contrast, in the presence of ATR-107, no pSTAT3 signal was observed in the nucleus by looking at the cells using ImageStream, agreeing with the lack of nuclear translocation.

Interestingly, ATR-107 inhibited the signal and the mechanism of inhibition was shown to be non-competitive. This mechanism of inhibition was demonstrated in both T and B cells. The data suggest that ATR-107 binding to the IL-21R is initially competitive with IL-21. However, once the antibody binds to the receptor, IL-21 cannot displace it from the receptor, thus, ATR-107 behaves like a functional non-competitive agent. This phenomenon is likely due to the long k_off_ (2.91 x 10^4^/s, t_1/2_: 39 min) of this antibody [14] which generates slow-reversible binding, an inhibitory feature of ATR-107. The non-competitive nature of inhibition demonstrated by ATR-107 may have important pharmacological consequences, since this mechanism could produce pharmacodynamic effects much longer that what is expected based on the pharmacokinetic characteristic of the molecule. Indeed, initial clinical data is suggestive of this effect where full receptor occupancy was observed for at least 42 days after a single administration of ATR-107 [19], an effect that was also demonstrated using pSTAT3 as a pharmacodynamic biomarker. These results in humans confirmed the previous observation of a 5–13 week pharmacodynamic efficacy observed in non-human primates using the IL-2R transcript as a PD biomarker [15].

Another important piece of information obtained from this study is that it is feasible to consider that the antibody binds the receptor initially in the cells that are circulating in the blood compartment and these cells may either not be able to infiltrate to the inflamed site or if they do, they may help transport the antibody to the inflamed tissues increasing its concentration at the site where it is needed. These possibilities will need to be demonstrated in further studies. Recent studies (Palandra et al. submitted Analytical Chemistry 2013) using a very specific mass spectrometric method to detect and quantify IL-21 demonstrated that IL-21 is not present in the circulation and rather is detected in inflamed tissues. This observation suggests that the antibody needs to inhibit the IL-21R at the inflamed site to be effective.

In summary, the results reported have two clinical applications. First, the functional pSTAT3 assay correlates well with the IL-21 receptor occupancy, suggesting these two assays could be used to support each other in assessing the pharmacology of ATR-107 in patients. Second, the pSTAT3 assay helped to discover the non-competitive feature of ATR-107, which provides important new information to predict ATR-107 effective dose selection. The data shows ATR-107 can completely block IL-21 induced STAT3 phosphorylation, regardless of the IL-21 concentration. Thus, for therapeutic purposes, the amount of IL-21R present on the cells at the inflamed site, but not the IL-21 levels is key to predicting the ATR-107 dose.

An important caveat from our studies is that all the assays reported here were done using blood from healthy donors. Levels of IL-21 and IL-21R could be quite different in patients with autoimmune diseases. For example, in lupus, the IL-21 producing T cells have been shown to be increased [21,22], while the IL-21R expression on B cells appears to be decreased [23] or unchanged [24]. Also, there may be changes in the sensitivity of the receptor activation as has been suggested for IL-21 in B cells in systemic lupus [24]. Thus, it is conceivable that the responses of immune cells to IL-21 and to ATR-107 blockade could be different in patients than in healthy controls. Thus, to accurately choose the therapeutic doses, the assay will need to be validated using blood from patients with the specific disease being targeted.

## Conclusions

Overall, this study shows that pSTAT3 can be used as a target biomarker to understand the inhibitory properties of ATR-107 in humans and it may help with the translation of preclinical information and dose selection towards ATR-107 clinical efficacy.

## Abbreviations

TM: Triple Mutant; STAT: Signal Transducer and Activator of Transcription; R: Receptor; Ab: Antibody; CD: Cluster of Differentiation; Ctr: Control; pSTAT: Phosphorylated Signal Transducer and Activator of Transcription; NK: Natural Killer; MFI: Mean Fluorescence Intensity; JAK: Janus Kinase; IL: Interleukin; Fc: Fragment Crystallizable; FITC: Fluorescein Isothiocyanate; IC50: 50% Inhibitive Concentration; Cat#: catalog number.

## Competing interests

All authors are employees of Pfizer Inc.

## Authors’ contributions

MZ participated in the design of the study, and carried out the ex vivo pSTAT3 whole blood assay, analyzed the data and drafted the manuscript. SPW performed pSTAT3 and the imaging flow cytometry assay. THL involved in the conception of the study. DAW performed the receptor occupancy assay. JBC participated in design of the study, analyzed the data and drafted the manuscript. JLM conceived the study, participated in its design, review the data and helped with the manuscript preparation. All authors read and approved the final manuscript.
